# Disruption of VGLUT1 in Cholinergic Medial Habenula Projections Increases Nicotine Self-Administration

**DOI:** 10.1523/ENEURO.0481-21.2021

**Published:** 2022-01-05

**Authors:** Elizabeth A. Souter, Yen-Chu Chen, Vivien Zell, Valeria Lallai, Thomas Steinkellner, William S. Conrad, William Wisden, Kenneth D. Harris, Christie D. Fowler, Thomas S. Hnasko

**Affiliations:** 1 Department of Neurosciences, University of California, San Diego, La Jolla, CA 92093; 2 Department of Neurobiology and Behavior sciences, University of California, Irvine, Irvine, CA 92697; 3 Department of Life Sciences and UK Dementia Research Institute, Imperial College London, London SW7 2AZ, United Kingdom; 4 Institute of Neurology, University College London, London WC1N 3BG, United Kingdom; 5 Research Service VA San Diego Healthcare System, San Diego, CA 92161

**Keywords:** acetylcholine, corelease, glutamate, interpeduncular nucleus, medial habenula, nicotine

## Abstract

Cholinergic projections from the medial habenula (MHb) to the interpeduncular nucleus (IPN) have been studied for their complex contributions to nicotine addiction and have been implicated in nicotine reinforcement, aversion, and withdrawal. While it has been established that MHb cholinergic projections corelease glutamate, no direct evidence has demonstrated a role for this glutamate projection in nicotine consumption. In the present study, a novel floxed *Slc17a7* [vesicular glutamate transporter 1 (VGLUT1)] mouse was generated and used to create conditional knock-out (cKO) mice that lack VGLUT1 in MHb cholinergic neurons. Loss of *Slc17a7* expression in ventral MHb cholinergic neurons was validated using fluorescent *in situ* hybridization, and immunohistochemistry was used to demonstrate a corresponding reduction of VGLUT1 protein in cholinergic terminals in the IPN. We also used optogenetics-assisted electrophysiology to evoke EPSCs in IPN and observed a reduction of glutamatergic currents in the cKO, supporting the functional disruption of VGLUT1 in MHb to IPN synapses. cKO mice exhibited no gross phenotypic abnormalities and displayed normal thigmotaxis and locomotor behavior in the open-field assay. When trained to lever press for food, there was no difference between control and cKO. However, when tested in a nicotine self-administration procedure, we found that the loss of VGLUT1-mediated glutamate corelease led to increased responding for nicotine. These findings indicate that glutamate corelease from ventral MHb cholinergic neurons opposes nicotine self-administration, and provide additional support for targeting this synapse to develop potential treatments for nicotine addiction.

## Significance Statement

Excitatory projections from the medial habenula (MHb) to interpeduncular nucleus (IPN) have been studied for their role in mediating the aversive properties of nicotine and nicotine intake behaviors. Although these projections are known to corelease acetylcholine and glutamate, the present study is the first investigation of a function for this glutamate signaling in nicotine consumption. We demonstrate that a loss of vesicular glutamate transporter 1 (VGLUT1) from cholinergic MHb neurons promotes increased nicotine self-administration in mice. Thus, we outline a role for glutamate release from MHb cholinergic projections in mediating the aversive properties of nicotine, expanding our knowledge of the neurobiology underlying nicotine consumption and identifying a possible substrate for therapeutic intervention.

## Introduction

Despite decades of research demonstrating the negative consequences of smoking and emerging evidence on harmful effects of electronic cigarettes, the use of tobacco products persists. In the United States, nicotine use among adolescents has increased in recent years. For instance, in 2019, 23% of middle and high school students reported use of a nicotine-containing product in the past 30 d, up from 9% in 2014 (Kasza et al., 2017; Wang et al., 2019). As the main psychoactive and addictive compound, nicotine remains at the forefront of this continuing public health crisis (Dani and Heinemann, 1996; Mansvelder and McGehee, 2002; Cooper and Henderson, 2020; Wittenberg et al., 2020).

Nicotine mediates its psychoactive effect by acting on nicotinic acetylcholine receptors (nAChRs) in the brain. Within the mesolimbic circuit, including ventral tegmental area dopamine neuron projections to nucleus accumbens, activation of nAChRs contribute to the rewarding effect of nicotine (Calabresi et al., 1989; Pontieri et al., 1996; Mansvelder and McGehee, 2000; Pons et al., 2008; Liu et al., 2012; Peng et al., 2017; Grieder et al., 2019; Akers et al., 2020). Conversely, the excitatory projection from medial habenula (MHb) to interpeduncular nucleus (IPN) has been identified as a key substrate on which nicotine actions contribute to nicotine aversion (Salas et al., 2009; Fowler and Kenny, 2011, 2014; Fowler et al., 2011; Frahm et al., 2011, 2015; Antolin-Fontes et al., 2015, 2020; Tuesta et al., 2017; Elayouby et al., 2021).

Both MHb and IPN express high levels of several nAChR subunits which modulate excitability and neurotransmission within this projection (Dineley-Miller and Patrick, 1992; Marks et al., 1992; McGehee et al., 1995; Perry et al., 2002; Salas et al., 2004; Shih et al., 2014). Mice lacking α5-containing nAChRs self-administered significantly more nicotine at high (typically aversive) doses, an effect which was normalized by viral expression of α5 nAChR subunit in the MHb or IPN, suggesting that α5-containing nAChRs in this circuit are necessary for nicotine aversion (Fowler et al., 2011). Additionally, overexpression of β4 nAChR subunit led to enhanced MHb activity and a strong aversive response to nicotine, which was abolished by disruption of the α5 nAChR subunit in MHb (Frahm et al., 2011). More recently, it has been shown that knock-down of the α3 nAChR subunit in either the MHb or IPN increased nicotine intake in rats (Elayouby et al., 2021). Together these data suggest that nAChRs containing the α5, α3, and β4 subunits mediate aversive signaling through MHb→IPN. Further, IPN projections to laterodorsal tegmentum (LDTg) are also strongly modulated by nicotine, and inhibiting this projection is sufficient to block nicotine aversion (Wolfman et al., 2018). Together, these findings establish the importance of MHb projections to IPN in modulating intake of nicotine and encoding its aversive properties.

MHb-IPN projections are topographically organized, with ventral MHb cholinergic projections targeting central IPN and Substance P-containing projections from dorsal MHb targeting lateral IPN (Herkenham and Nauta, 1979; Contestabile et al., 1987; Quina et al., 2017). The cholinergic ventral MHb expresses nAChRs and has been particularly implicated in nicotine aversion (Dineley-Miller and Patrick, 1992; Marks et al., 1992; Perry et al., 2002; Fowler et al., 2011; Frahm et al., 2011; Shih et al., 2014; Harrington et al., 2016). However, these neurons also express vesicular glutamate transporter 1 (VGLUT1) and can thus corelease both ACh and glutamate (Ren et al., 2011; Aizawa et al., 2012; Frahm et al., 2015), raising the question of what the glutamate signal from MHb may contribute to nicotine intake. To address this question, we made a new conditional VGLUT1 mouse line and used it to generate conditional knock-out (cKO) mice that lack VGLUT1 in ventral MHb cholinergic neurons. We showed that cKO mice have reduced glutamate transmission in MHb projections to IPN and that cKO mice displayed increased intravenous nicotine self-administration, consistent with a role for VGLUT1-mediated glutamate corelease at this circuit in opposing nicotine intake.

## Materials and Methods

### Animals

Mice were used in accordance with the University of California, San Diego and the University of California, Irvine Institutional Animal Care and Use Committees. BAC transgenic Kiaa1107-Cre mice were obtained from GENSAT through the MMRRC (#034692-UCD). Kiaa-Cre mice were bred hemizygously with C57Bl/6J wild-type mice (The Jackson Laboratory, 000664). VGLUT2-IRES-Cre (*Slc17a6^Cre^*) knock-in mice were ordered from The Jackson Laboratory (#028863) and bred homozygously or to C57BL6/J wild-type mice. All experiments were done in adult mice (aged more than eight weeks) and in both males and females in approximately equal proportion.

### VGLUT1 conditional allele

To generate VGLUT1-floxed mice (*Slc17a7*^flox^), a targeting vector containing two loxP sites flanking *Slc17a7* exons 4–7 and an FRT-flanked neomycin (Neo) resistance cassette was electroporated into C57Bl/6-derived ES cells. Antibiotic (G418)-resistant colonies were selected, isolated, and amplified. The amplified clones were screened for homologous recombination at the *Slc17a7* locus by PCR. Southern blot analysis was used to confirm both 3′ and 5′ homologous recombination. Blastocysts were isolated from pregnant C57Bl/6J-Tyr^c-2J^/J (albino C57Bl/6) females, injected with one of six validated ES cell clones, and implanted into pseudo-pregnant females. Chimeric males were bred to C57Bl/6 females with constitutive expression of FLP recombinase to excise the Neo cassette in F1 offspring. F1 mice were crossed to C57Bl/6, excision was confirmed by PCR and Southern blotting, and these F2 mice were used to establish the VGLUT1 floxed line (*Slc17a7^flox^*). To generate cKO mice (*Kiaa^Cre^; Slc17a7^flox/flox^*), Kiaa^Cre^ mice were bred to homozygous *Slc17a7^flox/flox^* and resulting heterozygotes (*Kiaa^Cre^; Slc17a7^+/flox^*) were then bred to homozygous *Slc17a7^flox/flox^* mice. cKO mice used for these studies were generated from eight different breeder cages using 16 breeder mice. Mice were group housed and maintained on a 12/12 h light/dark cycle. Food and water were available *ad libitum* except where noted.

### Stereotactic surgery

For intracranial injections, mice (more than four weeks) were deeply anesthetized with isoflurane, placed in a stereotaxic frame (Kopf), and bilaterally injected with AAV1-Ef1a-DIO-ChR2:mCherry (2 × 10^12^, UNC Gene Therapy Center) into the MHb (LM = −1.15, AP = −1.58, DV = −2.42 and −2.00, 20° angle; right: LM = +0.95, AP = −1.58, DV = −2.42 and −2.00, 20° angle; mm relative to *Bregma*). Two 150-nl aliquots were given per hemisphere at 100 nl/min using pulled glass pipettes (Nanoject III, Drummond Scientific). Analgesic was given before and at least 1 d after surgery (carprofen, Zoetis, 5 mg/kg, s.c.). Mice were monitored daily for 5 d after surgery and allowed to recover for at least 21 d before histologic processing or 28 d before electrophysiological recordings.

### Immunohistochemistry

Mice were deeply anesthetized with pentobarbital (200 mg/kg, s.c., VetOne) and transcardially perfused for 2 min with ice-cold PBS then for 8 min with ice-cold 4% paraformaldehyde (PFA) at a rate of 5–6 ml/min. Brains were prepared as previously described (Faget et al., 2018). Primary antibodies used: DsRed (rabbit, 1:2000, Takara Bio, RRID:AB_10013483), VGLUT1 (guinea pig, 1:2000, Synaptic Systems, RRID:AB_887878), VGLUT2 (rabbit, 1:1000, Synaptic Systems, RRID:AB_887883), choline acetyltransferase (ChAT; goat, 1:200, Millipore, RRID:AB_2079751). Secondary antibodies used (5 μg/ml, Jackson ImmunoResearch): Alexa Fluor 488 donkey anti-goat (705-545-147, RRID:AB_2336933), Alexa Fluor 488 donkey anti-guinea pig (706-545-148, RRID:AB_2340472), Alexa Fluor 594 donkey anti-guinea pig (706-585-148, RRID:AB_2340474), Alexa Fluor 594 donkey anti-rabbit (711-585-152, RRID:AB_2340621), Alexa Fluor 647 donkey anti-rabbit (711-605-152, RRID:AB_2492288), Alexa Fluor 647 donkey anti-goat (705-605-147, RRID:AB_2340437). Images were captured using a Zeiss AxioObserver Z1 epifluorescence microscope (10 × 0.45 NA, 20 × 0.75 NA, or 63 × 1.4 NA objective) and Zen software. Zen software was used to set levels for each channel, and these parameters were applied identically across sections. Adobe Photoshop was used to delineate the boundaries of the IPN. Densitometry was done with Fiji/ImageJ using the Measure analysis tool. No background subtraction was performed. Two to three sections were quantified per mouse; one to three sections excluded per mouse because of tissue damage. No mice were excluded from analysis following immunohistochemistry.

### Fluorescent *in situ* hybridization

Mice were deeply anesthetized with pentobarbital before cervical dislocation. Brains were prepared as previously described (Faget et al., 2018). *In situ* hybridization was done using RNAscope Multiplex Fluorescent Assay (Advanced Cell Diagnostics) according to manufacturer specification. *Slc17a7* (503511), *ChAT* (408731-C2), and *Cre* (312281-C3) were coupled to Atto550, Alexa Fluor 647, and Alexa Fluor 488, respectively, and counterstained with DAPI. Images were captured using a Zeiss AxioObserver Z1 epifluorescence microscope and processed with Zen software as described above. Adobe Photoshop was used to outline the MHb and densitometry was done with the Fiji/ImageJ Measure analysis tool. No background subtraction was performed. One to four sections were used per mouse; one to four sections excluded per mouse because of tissue damage and/or signal indicative of cutting or labeling artifacts. No mice were excluded from analysis.

### Open-field behavior

Mice were placed in an Open Field (30 m) measuring 50 × 50 cm and their activity was recorded and analyzed using AnyMaze software (San Diego Instruments). The field was cleaned with 70% ethanol between sessions. The field was segmented into a 5 × 5 grid, with the innermost nine squares designated as the center.

### Operant behavior

Operant testing and self-administration studies were performed by experimenters blind to genotype. Mice were fed 2–4 g per mouse per day to achieve mild food-restriction to 85–90% of their free-feeding weight and were then trained to lever press for food pellets (grain-based, 20 mg, 5TUM, TestDiet) on a two-lever operant task across ascending fixed ratio (FR) schedules from one up to five lever presses, as previously described (Fowler and Kenny, 2011). At the start of the session, both levers were extended into the chamber and were present throughout the 1 h session. Responses on the active lever that met the FR criteria resulted in the delivery of a food pellet, which was paired with a cue light for a 20-s time-out period, resulting in the final reinforcement schedule of FR5TO20 for food training sessions 4–7. Responses on the inactive lever were recorded but had no scheduled consequences. Testing was conducted 6–7 d per week, and behavioral responses were recorded with a MedPC interface (Med Associates). Thereafter, subjects were anesthetized (isoflurane) and catheterized as previously described (Fowler and Kenny, 2011; Chen et al., 2018). The catheter tubing was passed subcutaneously from the animal’s back to the right jugular vein, a 1 cm length of catheter tip was inserted into the vein and tied with surgical silk suture. Following surgery animals were allowed ≥48 h to recover, and were then provided 1-h access to reestablish food responding under the FR5TO20 sec schedule until the criteria of >30 pellets/session were again achieved. Mice were then transitioned to respond for intravenous nicotine self-administration in lieu of food using the same FR5TO20 sec, 1 h daily sessions, 6–7 d per week, at the training dose of nicotine (0.03 mg/kg per infusion) for 8 d. For all doses, nicotine (0.03 ml per infusion volume) was delivered through tubing into the intravenous catheter by a Razel syringe pump (Med Associates). Based on prior findings (Fowler and Kenny, 2011; Fowler et al., 2011), mice typically achieve stable responding for nicotine after ∼5 d of acquisition, which can be evidenced by <20% variability in responding between consecutive sessions. All mice were provided access to the acquisition dose of nicotine for 8 d to allow for consistency in the total number of sessions, although many subjects acquired stable responding before 8 d. After achieving stable responding on the 0.03 mg/kg per infusion dose, mice were transitioned to the moderate dose of 0.1 mg/kg per infusion nicotine for 5 d. This dose results in a similar levels of drug intake as that found at higher doses with behavioral titration via self-administration and was used to further establish baseline responding in between access to each subsequent varying dose (Fowler and Kenny, 2011). Next, the mice were provided access to either the low 0.01 mg/kg per infusion or high 0.4 mg/kg per infusion dose for 5 d, and then reestablished at baseline on 0.1 mg/kg per infusion for at least 2 d, and thereafter given access to the counterbalanced doses of either 0.01 or 0.4 mg/kg per infusion for an additional 5 d. Following reestablishing baseline for at least 2 d, the mice were provided access to respond for saline vehicle. The mean of the final 3 d on each dose was calculated for each subject. Catheters were flushed daily with physiological sterile saline solution (0.9% w/v) containing heparin (100 units/ml). Catheter integrity was verified with the ultra-short-acting barbiturate anesthetic Brevital (2%, methohexital sodium, Eli Lilly) at the end of the study. One male mouse was excluded from nicotine self-administration behavior because of excessive barbering injuries and one female excluded because of a leaky catheter.

### Electrophysiological recordings

Recordings were performed by experimenters blind to genotype. Recordings were performed on adult mice (7–12 weeks) as previously described (Zell et al., 2020). mCherry-labeled MHb terminals were visualized by epifluorescence and visually guided patch recordings were made using infrared-differential interference contrast (IR-DIC) illumination (Axiocam MRm, Examiner.A1, Zeiss). ChR2 was activated by flashing blue light (473 nm) through the light path of the microscope using a light-emitting diode (UHP-LED460, Prizmatix) under computer control. Neurons were held in voltage-clamp at −60 mV to record EPSCs in whole-cell configuration and single-pulse photostimuli (5-ms or 1-s pulse width) were applied every 45 s, and 10 photo-evoked currents were averaged per neuron per condition. Stock solutions of DNQX (10 mm in DMSO, Sigma) and mecamylamine hydrochloride (10 mm, Tocris) were diluted 1000-fold in artificial CSF (ACSF) and bath applied at 10 μm. Current sizes were calculated by using peak amplitude from baseline. Identification of glutamatergic or cholinergic currents relied primarily on established kinetic properties, with pharmacology used to confirm in a subset of cells (Ren et al., 2011; Frahm et al., 2015).

### Statistics

Data analysis was done using GraphPad Prism v9. Data were analyzed using *t* test corrected for multiple comparisons (Bonferroni–Sidak), unpaired *t* test, mixed-effects analysis (Sidak *post hoc*; Table 1). Unless otherwise stated, data presented represent mean, symbols represent individual values, and error bars represent standard error of the mean.

**Table 1:** Statistics table

Figure	Type of test	Statistical data
1*F*	*t* test (Bonferroni–Sidak)	Dorsal: *t*_(4)_ = 0.004, *p*(adj) = 0.99; ventral: *t*_(4)_ = 0.27, *p*(adj) = 0.96
1*G*	*t* test (Bonferroni–Sidak)	Dorsal: *t*_(4)_ = 1.0, *p*(adj) = 0.61; ventral: *t*_(4)_ = 6.4, *p*(adj) = 0.006
1*H*	*t* test (Bonferroni–Sidak)	Dorsal: *t*_(4)_ = 0.79, *p*(adj) = 0.72; ventral: *t*_(4)_ = 0.81, *p*(adj) = 0.71
2*B*	*t* test (Bonferroni–Sidak)	Central: *t*_(6)_ = 0.89, *p*(adj) = 0.65; lateral: *t*_(6)_ = 1.2, *p*(adj) = 0.50
2*C*	*t* test (Bonferroni–Sidak)	Central: *t*_(6)_ = 5.2, *p*(adj) = 0.004; lateral: *t*_(6)_ = 0.30, *p*(adj) = 0.95
2*D*	*t* test (Bonferroni–Sidak)	Central: *t*_(6)_ = 0.31, *p*(adj) = 0.94; lateral: *t*_(6)_ = 0.14, *p*(adj) = 0.99
4*C*	Unpaired *t* test	*t*_(14)_ = 4.0, *p* = 0.001
4*D*	Unpaired *t* test	*t*_(13)_ = 1.6, *p* = 0.14
5*A*	Mixed-effects analysis (Sidak)	Main effect of segment, *F*_(2.6,36)_ = 30, *p* < 0.0001; genotype, *F*_(1,14)_ = 0.12, *p* = 0.73; segment × genotype interaction, *F*_(5,70)_ = 2.0, *p* = 0.084
5*B*	Mixed-effects analysis (Sidak)	Main effect of segment, *F*_(3.4,47)_ = 1.1, *p* = 0.38; genotype, *F*_(1,14)_ = 0.14, *p* = 0.71; segment × genotype interaction, *F*_(5,70)_ = 0.19, *p* = 0.97
5*C*	Mixed-effects analysis (Sidak)	Main effect of session, *F*_(6,108)_ = 72, *p* < 0.0001; genotype, *F*_(1,108)_ = 0.10, *p* = 0.75; lever, *F*_(1,18)_ = 243, *p* < 0.0001; session × genotype, *F*_(6,108)_ = 0.90, *p* = 0.50; session × lever, *F*_(6,108)_ = 74, *p* < 0.0001; genotype × lever, *F*_(1,108)_ = 0.026, *p* = 0.87; session × genotype × lever, *F*_(6,108)_ = 1.3, *p* = 0.25
5*D*	Mixed-effects analysis (Sidak)	Main effect of session, *F*_(7,112)_ = 38, *p* < 0.0001; genotype, *F*_(1,112)_ = 11, *p* = 0.001; lever, *F*_(1,16)_ = 111, *p* < 0.0001; session × genotype, *F*_(7,112)_ = 1.8, *p* = 0.099; session × lever, *F*_(7,112)_ = 33, *p* < 0.0001; genotype × lever, *F*_(1,112)_ = 8.5, *p* = 0.004; session × genotype × lever, *F*_(7,112)_ = 0.95, *p* = 0.47
5*E*	Unpaired *t* test	*t*_(16)_ = 2.3, *p* = 0.035
5*F*	Mixed-effects analysis (Sidak)	Main effect of dose, *F*_(4,64)_ = 23, *p* < 0.0001; genotype, *F*_(1,16)_ = 2.3, *p* = 0.15; dose × genotype interaction, *F*_(4,64)_ = 3.5, *p* = 0.012

**Figure 1.**
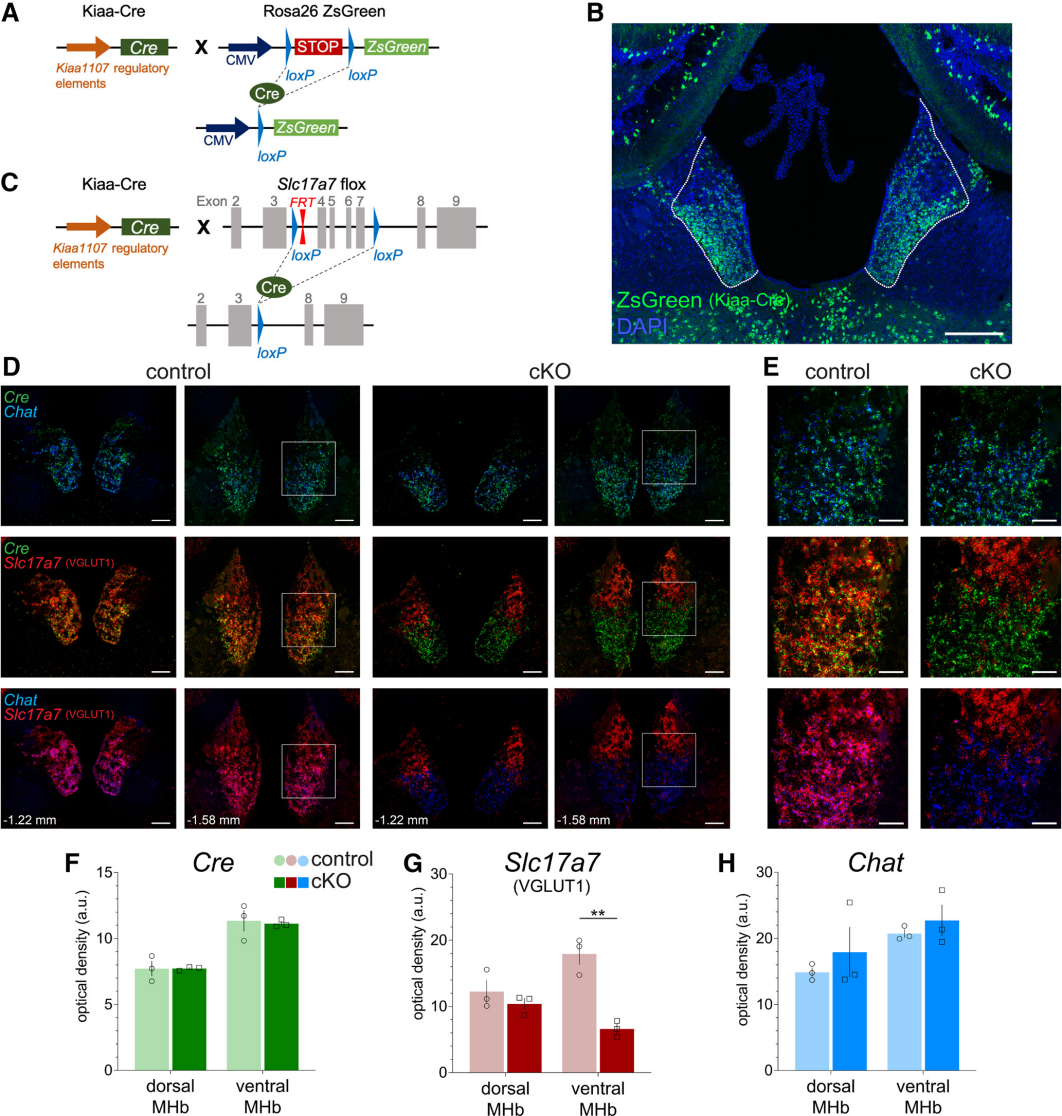
cKO of *Slc17a7* (VGLUT1) from cholinergic neurons in MHb. ***A***, Schematic of *Kiaa^Cre^*; *Rosa26^ZSGreen^* reporter mouse line with Cre expression driven by *Kiaa1107* regulatory elements and ZsGreen expression dependent on Cre recombination. ***B***, Native ZsGreen fluorescence counterstained with DAPI in MHb (outlined); scale bar: 200 μm. ***C***, Schematic of Cre recombination of the floxed *Slc17a7* (VGLUT1) locus in the cKO (*Kiaa^Cre^*; *Slc17a7^flox/flox^*) mouse line. ***D***, Fluorescent *in situ* hybridization of *Cre*, *Chat*, and *Slc17a7* expression in MHb of control and cKO mice at two bregma points; scale bar: 100 μm. ***E***, Higher-magnification images from white squares in ***D***; scale bar: 50 μm. Densitometric quantification (without background subtraction) in ventral and dorsal MHb of (***F***) *Cre*, (***G***) *Slc17a7* (VGLUT1), and (***H***) *Chat* signals. Only *Slc17a7* was significantly reduced in ventral MHb of cKO (**p = 0.006); *n* = 3 mice per group.

**Figure 2.**
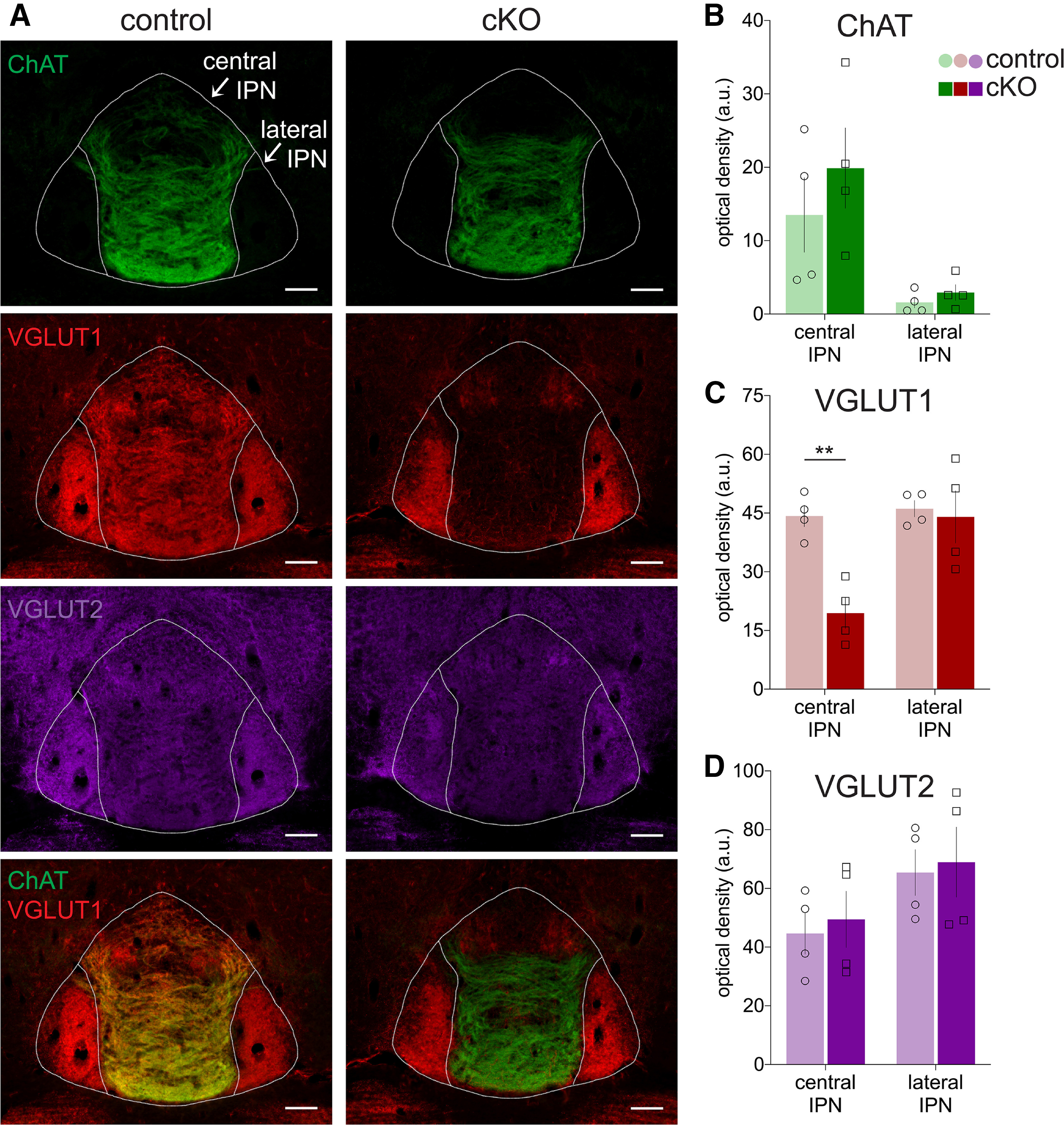
Loss of VGLUT1 in central IPN of cKO mice. ***A***, Immunohistochemistry for ChAT, VGLUT1, and VGLUT2 in the IPN of control (*Slc17a7^flox^*) and cKO mice (*Kiaa^Cre^*; *Slc17a7^flox/flox^*); bottom row shows ChAT and VGLUT1 merge; scale bar: 100 μm. Densitometric quantification (without background subtraction) in central and lateral IPN of (***B***) ChAT, (***C***) VGLUT1, and (***D***) VGLUT2 signals. Only VGLUT1 was significantly reduced in cKO and only in the central IPN (**p = 0.0040); *n* = 4 mice per group.

## Results

### Generation of *Slc17a7* cKO from ventral MHb

To target cholinergic neurons of the MHb we used the Kiaa1107-Cre (*Kiaa^Cre^*) transgenic line that has been used previously to disrupt ChAT expression in MHb (Frahm et al., 2015). The functional expression of *Cre recombinase* driven by *Kiaa1107* regulatory elements was first validated by crossing to the Ai6 ZsGreen reporter (Madisen et al., 2010) to generate *Kiaa^Cre^; Rosa26^ZsGreen^* mice (Fig. 1*A*). Robust ZsGreen fluorescence was seen in MHb, with densest expression observed in ventral (basolateral and basomedial) MHb (Fig. 1*B*). Importantly, we also observed ZsGreen expression in VGLUT1-rich regions of cortex and hippocampus, but although some of these structures express VGLUT1, they are not known to project to IPN (expression pattern for the founder line KJ227 can be viewed throughout the rostral-caudal extent of brain at gensat.org).

To disrupt VGLUT1 expression, we generated a novel mouse line carrying a VGLUT1 conditional allele (*Slc17a7^flox^*) with exons 4–7 flanked by loxP sites (Fig. 1*C*). We next crossed *Slc17a7^flox^* mice to *Kiaa^Cre^* to generate the VGLUT1 cKO (*Kiaa^Cre^; Slc17a7^flox/flox^*). We generated an RNAscope probe targeting exons 4–7 of *Slc17a7* and used this together with probes against a cholinergic marker (*Chat*) and *Cre recombinase* on sections from cKO and control (*Kiaa^Cre^*) mice (Fig. 1*D*,*E*). The pattern of *Cre* expression was identical for both genotypes and similar to the pattern observed in the ZsGreen reporter cross, with robust expression in ventral MHb. *Chat* appeared unchanged across genotype and showed high overlap with *Cre.* Consistent with other reports, *Chat* expression was largely restricted to ventral MHb (Oh et al., 1992; Trifonov et al., 2009; Aizawa et al., 2012; Görlich et al., 2013).

Also consistent with other previous reports, *Slc17a7* (VGLUT1) was expressed throughout the MHb in controls (Fremeau et al., 2001; Varoqui et al., 2002; Barroso-Chinea et al., 2007; Aizawa et al., 2012). However, the cKO showed a markedly different pattern (Fig. 1*D*,*E*). In cKO mice, *Slc17a7* (VGLUT1) expression was significantly reduced in ventral MHb (*t*_(4)_ = 6.4, *p*(adj) = 0.006), but was intact in dorsal (apical) MHb (*t*_(4)_ = 1.0, *p*(adj) = 0.61; Fig. 1*G*). There was no difference in *Cre* expression in either dorsal (*t*_(4)_ = 0.004, *p*(adj) = 0.99) or ventral (*t*_(4)_ = 0.27, *p*(adj) = 0.96) MHb (Fig. 1*F*). There was also no significant difference in *Chat* expression in dorsal (*t*_(4)_ = 0.79, *p*(adj) = 0.72) or ventral (*t*_(4)_ = 0.81, *p*(adj) = 0.71) MHb between groups (Fig. 1*H*). *Chat* expression was used to delineate the boundary between dorsal and ventral MHb. These results indicate that our cKO successfully and selectively disrupted *Slc17a7* expression from Cre-expressing neurons in ventral MHb.

We next used immunohistochemistry to examine the expression of presynaptic cholinergic and glutamatergic markers in the IPN, the major projection target of MHb (Fig. 2*A*). While the expression of ChAT was unaffected in the cKO (Fig. 2*B*), the pattern of VGLUT1-labeled fibers was markedly different depending on genotype and subregion. cKO mice had a significant reduction of VGLUT1 expression compared with controls in central IPN (*t*_(6)_ = 5.2, *p*(adj) = 0.004), but no difference in VGLUT1 between genotypes was observed in lateral IPN (*t*_(6)_ = 0.30, *p*(adj) = 0.95; Fig. 2*C*). Together, these data are concordant with our RNAscope data and demonstrate the selective disruption of VGLUT1 from cholinergic MHb inputs that target the central region of the IPN, which includes the caudal, dorsomedial, intermediate, and rostral subnuclei.

We also examined VGLUT2-labeled fibers in the IPN to test whether the loss of VGLUT1 led to changes in VGLUT2 expression. We detected no significant difference in VGLUT2 expression between genotypes in either central IPN (*t*_(6)_ = 0.41, *p*(adj) = 0.91) or lateral IPN (*t*_(6)_ = 0.25, *p*(adj) = 0.96; Fig. 2*D*). These data argue against compensatory change in VGLUT2 expression following loss of VGLUT1 from cholinergic neurons in MHb.

### Expression of *Slc17a6* (VGLUT2) in IPN-projecting MHb neurons

**Figure 3.**
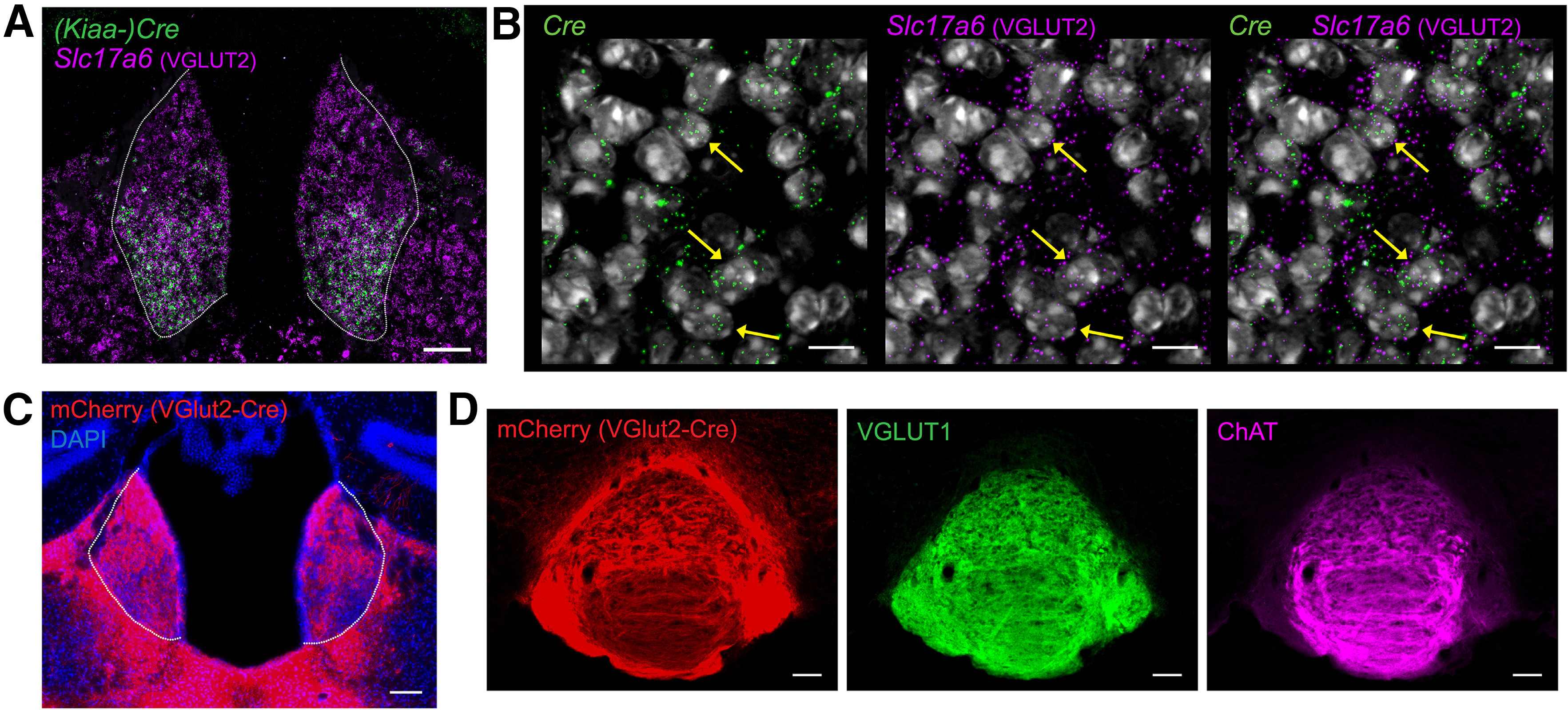
VGLUT2-expressing projections from MHb to IPN. ***A***, Fluorescent *in situ* hybridization from *Kiaa^Cre^* mouse showing *Cre* and *Slc17a6* expression in the MHb (outlined); scale bar: 100 μm. ***B***, High-resolution image showing expression of *Cre*, *Slc17a6* (VGLUT2), and DAPI in MHb of *Kiaa^Cre^* mouse; scale bar: 10 μm. Yellow arrows indicate some of the cells containing both *Cre* and *Slc17a6* mRNA. ***C***, Image of MHb from *Slc17a6^Cre^* (VGLUT2) mouse injected with AAV1-Ef1α-DIO-ChR2:mCherry bilaterally into the MHb (outlined); scale bar: 100 μm. ***D***, Immunohistochemistry of IPN from *Slc17a6^Cre^* (VGLUT2-Cre) mouse injected bilaterally with AAV1-Ef1α-DIO-ChR2:mCherry in MHb. VGLUT2^Cre^ MHb terminals in IPN represented by mCherry expression, stained with VGLUT1 and ChAT; scale bar: 100 μm.

The absence of *Slc17a7*/VGLUT1 expression in ChAT-expressing neurons of ventral MHb and central IPN provides strong evidence for loss of VGLUT1-mediated glutamatergic corelease from cholinergic MHb inputs in cKO mice. But while VGLUT1 has been implicated in mediating glutamate corelease from MHb cholinergic neurons (Ren et al., 2011; Aizawa et al., 2012; Frahm et al., 2015), there is also evidence that some MHb neurons express *Slc17a6*/VGLUT2 (Varoqui et al., 2002; Barroso-Chinea et al., 2007; Aizawa et al., 2012), consistent with the presence of VGLUT2-labeled fibers that we observed in IPN (Fig. 2*D*). To directly test whether *Kiaa^Cre^*-expressing MHb cells also express *Slc17a6* (VGLUT2) we used RNAscope. *Slc17a6* was observed throughout the MHb (Fig. 3*A*), including in the ventral MHb where it partially colocalized with Cre recombinase (*Kiaa^Cre^*; Fig. 3*B*).

The presence of MHb neurons coexpressing *Cre* and *Slc17a6* (VGLUT2) in cKO mice raised the question of whether this VGLUT2 population projected to IPN. We thus injected an adeno-associated virus (AAV) into the MHb of *Slc17a6^Cre^* (VGLUT2-Cre) mice (Vong et al., 2011) to Cre-dependently express Channelrhodpsin-2 fused to a fluorescent tag (ChR2:mCherry). Three weeks after surgery, mCherry expression was found in MHb, as well as in surrounding areas of lateral habenula (LHb) and paraventricular nucleus of the thalamus (PV; Fig. 3*C*). mCherry-expressing fibers, presumably axon terminals from MHb, were also present in both central and lateral IPN (Fig. 3*D*). These results indicate that at least some *Kiaa^Cre^* cholinergic neurons in MHb could express both VGLUT1 and VGLUT2, consistent with previous reports of VGLUT1/VGLUT2 coexpression in MHb (Aizawa et al., 2012; Frahm et al., 2015).

### Disruption of VGLUT1 from ventral MHb neurons decreased evoked glutamate currents in central IPN

**Figure 4.**
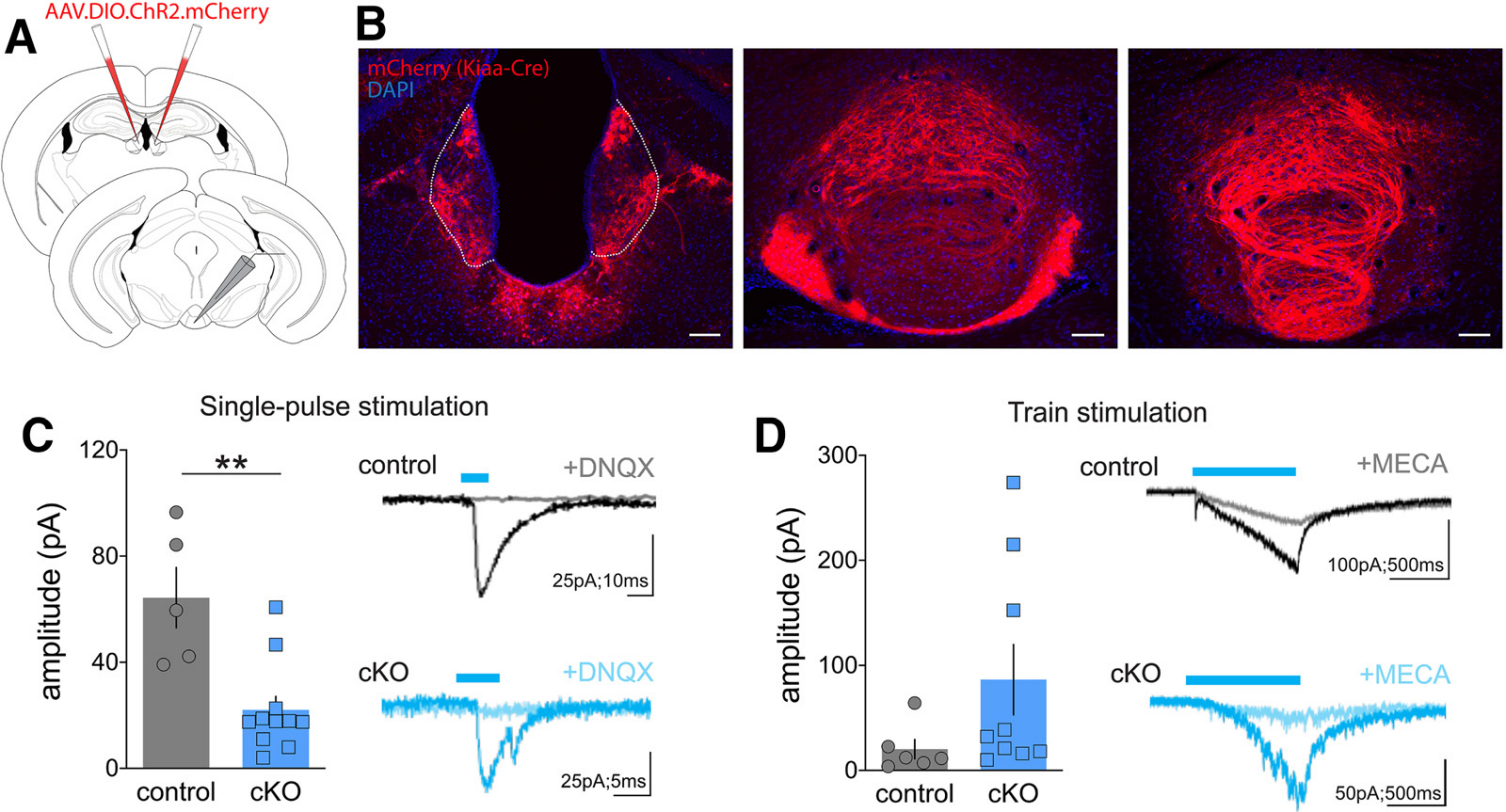
Reduced glutamate transmission from MHb to IPN in VGLUT1 cKO mice. ***A***, Schematic of electrophysiological preparation, with bilateral injections of AAV1-Ef1α-DIO-ChR2:mCherry in MHb of control (*Kiaa^Cre^*) or cKO (*Kiaa^Cre^*; *Slc17a7^flox/flox^*) mice. Slice recordings using optogenetic stimulation performed in the IPN 3+weeks after injection. ***B***, Images from control mouse of native Cre-dependent mCherry fluorescence in MHb (left) and fibers in IPN (center, right); scale bar: 100 μm. ***C***, Whole-cell recordings in IPN with single-pulse optogenetic stimulation of MHb terminals led to oEPSC amplitudes that were reduced in the cKO (left, **p = 0.001). Representative traces before and after DNQX in control (black) and cKO (blue; right). ***D***, oEPSC amplitude following train stimulation (1 s) did not differ significantly different between control and cKO groups (left). Representative traces before and after mecamylamine in control (black) and cKO (blue; right). Note that bars in panels ***C***, ***D*** represent mean ± SEM, individual cells are represented by gray circles (control) or blue squares (cKO).

We next tested how the loss of VGLUT1 from Cre-expressing ventral MHb cholinergic neurons affected glutamate transmission from terminals in central IPN. We expressed ChR2:mCherry in MHb as above, but now using *Kiaa^Cre^* and cKO mice that lack VGLUT1 in these neurons (Fig. 4*A*). ChR2:mCherry expression was observed in MHb and IPN (Fig. 4*B*); recordings were made from IPN neurons, with optogenetic stimulation of MHb terminals. Whole-cell voltage-clamp was used to assess optogenetic-evoked EPSCs (oEPSCs) in response to either a single pulse of blue light (5 ms) or train stimulation (5-ms pulses at 20 Hz for 1 s). Single-pulse stimulation evoked fast glutamatergic oEPSCs (Fig. 4*C*) that were significantly smaller but not eliminated in the cKO (unpaired *t* test*; t*_(14)_ = 4.0, *p* = 0.001). Residual currents were presumably because of expression of VGLUT2 in some of the Cre-expressing cholinergic neurons and were blocked by bath application of an AMPA-type glutamate receptor antagonist (mean EPSC before DNQX 46 ± 14 pA, after DNQX 1.5 ± 1.5 pA; *n* = 4). Train stimulation led to a mixed response that contained both faster glutamatergic, as well as slower cholinergic oEPSCs (Fig. 4*D*) that were blocked by a nAChR antagonist (mean EPSC before mecamylamine 126 ± 75 pA, after mecamylamine 24 ± 1.5 pA; *n* = 3). While the variability in responses to train stimulation appeared higher in the cKO, no significant difference in oEPSC amplitude was detected between genotypes in response to train stimulation (unpaired *t* test*; t*_(13)_ = 1.6, *p* = 0.14), suggesting cholinergic transmission in the cKO was largely intact, although more subtle functional changes cannot be excluded.

### Loss of MHb VGLUT1 increased nicotine self-administration

**Figure 5.**
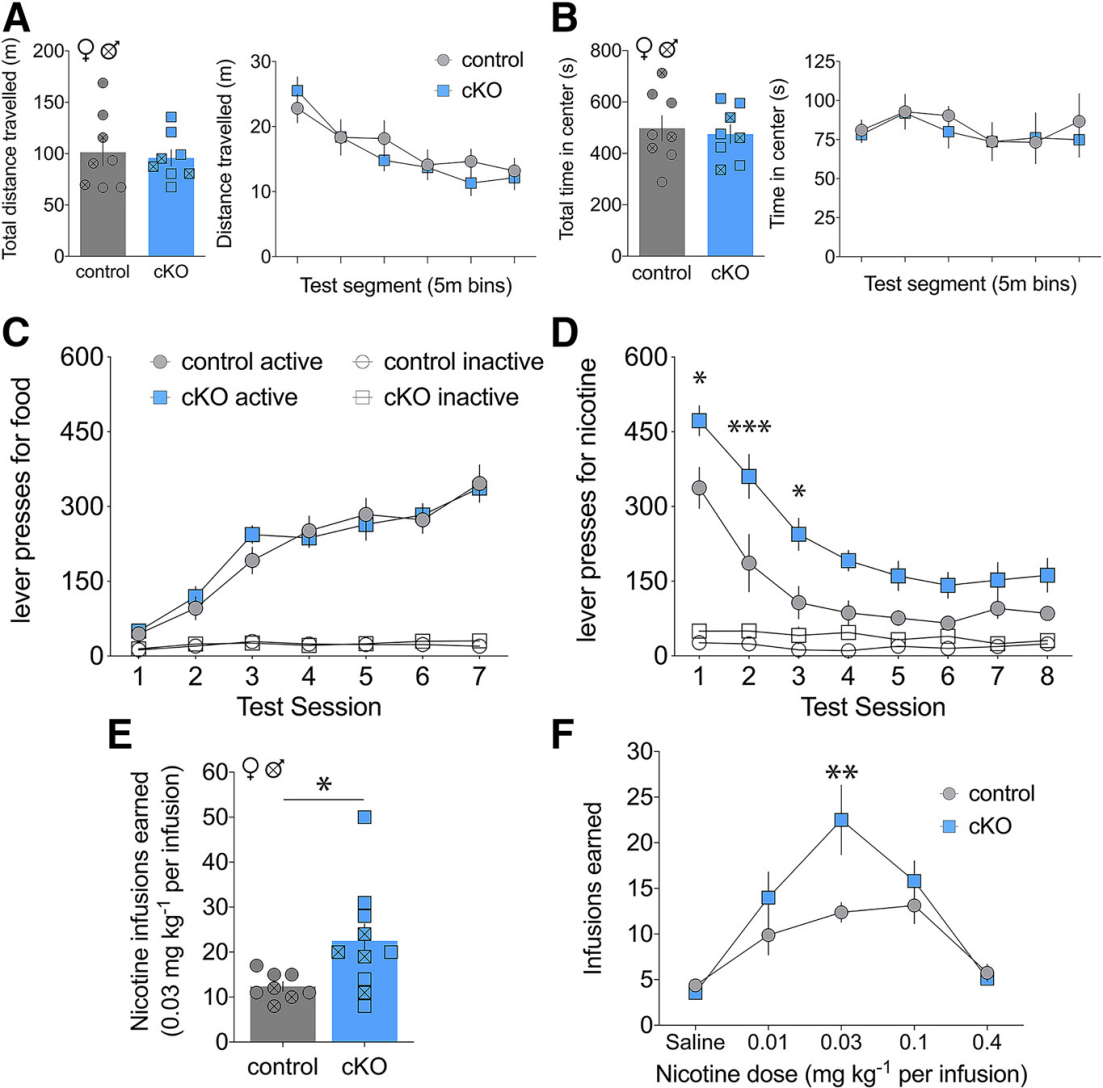
Increased nicotine self-administration in cKO mice. ***A***, Total distance traveled (left) and distance traveled across test segments (right) in open field assay showed no significant differences between genotype. ***B***, Total time in center (left) and time in center across test segments (right) did not differ between genotype. ***C***, Active and inactive lever presses during food training across test session did not differ by genotype. ***D***, Active and inactive lever presses for nicotine show increased self-administration for cKO mice (Sidak’s *p < 0.05, ***p < 0.001). ***E***, cKO mice earned more total nicotine infusions in first three nicotine test sessions (*t* test, *p < 0.05). ***F***, Nicotine infusions earned by control and cKO mice in dose–response paradigm (Sidak’s, **p < 0.005).

Prior studies have implicated MHb cholinergic signaling to IPN in the aversive effects of nicotine (Salas et al., 2009; Fowler and Kenny, 2011, 2014; Frahm et al., 2011, 2015; Harrington et al., 2016), but the contribution of glutamate corelease from this circuit had not been examined. We thus assessed the behavioral phenotype of littermate control and cKO mice. To test gross locomotor and exploratory behavior, we assessed mice in the open-field test. No significant differences were found between genotype in distance traveled (mixed-effects analysis; main effect of segment, *F*_(2.6,36)_ = 30, *p* < 0.0001; genotype, *F*_(1,14)_ = 0.12, *p* = 0.73; segment × genotype interaction, *F*_(5,70)_ = 2.0, *p* = 0.084; Fig. 5*A*) or time spent in center (main effect of segment, *F*_(3.4,47)_ = 1.1, *p* = 0.38; genotype, *F*_(1,14)_ = 0.14, *p* = 0.71; segment × genotype interaction, *F*_(5,70)_ = 0.19, *p* = 0.97; Fig. 5*B*).

Next, control and cKO mice were trained to lever press for food pellets and each reward delivery was paired with a cue-light for a 20-s time-out period (TO20). Across the initial three sessions, the FR schedule increased from one to five lever presses, then mice were maintained on an FR5 for an additional three sessions. No significant differences between genotypes were detected, suggesting intact operant learning and lever discrimination in cKO mice (mixed-effects analysis; main effect of session, *F*_(6,108)_ = 72, *p* < 0.0001; genotype, *F*_(1,108)_ = 0.10, *p* = 0.75; lever, *F*_(1,18)_ = 243, *p* < 0.0001; session × genotype, *F*_(6,108)_ = 0.90, *p* = 0.50; session × lever, *F*_(6,108)_ = 74, *p* < 0.0001; genotype × lever, *F*_(1,108)_ = 0.026, *p* = 0.87; session × genotype × lever, *F*_(6,108)_ = 1.3, *p* = 0.25; Fig. 5*C*).

After food training, intravenous catheters were implanted, and an acquisition dose of nicotine (0.03 mg/kg per infusion) was introduced at the established FR5TO20 schedule of reinforcement. As previously observed with this protocol (Fowler and Kenny, 2011), both groups pressed initially at a high rate similar to that observed with food reinforcement, which subsequently declined across sessions to a steady-state rate of nicotine self-administration (Fig. 5*D*). cKO mice engaged in consistently higher levels of nicotine self-administration across sessions at this dose (mixed-effects analysis; main effect of session, *F*_(7,112)_ = 38, *p* < 0.0001; genotype, *F*_(1,112)_ = 11, *p* = 0.001; lever, *F*_(1,16)_ = 111, *p* < 0.0001; session × genotype, *F*_(7,112)_ = 1.8, *p* = 0.099; session × lever, *F*_(7,112)_ = 33, *p* < 0.0001; genotype × lever, *F*_(1,112)_ = 8.5, *p* = 0.004; session × genotype × lever, *F*_(7,112)_ = 0.95, *p* = 0.47). Compared with controls, cKO mice earned significantly more total nicotine infusions in the first three test sessions (unpaired t test; *t*_(16)_ = 2.3, *p* = 0.035; Fig. 5*E*).

To assess across a range of nicotine doses, a dose–response was then performed. While both groups exhibited an inverted U-shaped dose–response, cKO mice showed a dose-dependent increase in nicotine consumption compared with controls and this effect was most pronounced at 0.03 mg/kg per infusion dose (mixed-effect analysis; main effect of dose, *F*_(4,64)_ = 23, *p* < 0.0001; genotype, *F*_(1,16)_ = 2.3, *p* = 0.15; dose × genotype interaction, *F*_(4,64)_ = 3.5, *p* = 0.012; Fig. 5*F*). Together, these results support the hypothesis that VGLUT1-mediated glutamate transmission from MHb to IPN opposes nicotine self-administration.

## Discussion

The present study provides direct evidence for the role of glutamate release from MHb cholinergic projections in opposing nicotine self-administration. Previous work on the role of MHb→IPN projections in nicotine consumption has focused principally on cholinergic transmission within this synapse. Indeed, nicotine facilitates glutamate release from MHb terminals by activating presynaptic nAChRs (McGehee et al., 1995; Girod et al., 2000). Knock-down of α5 nAChR in MHb led to increased nicotine consumption in mice, as did blocking glutamate transmission in IPN by microinjection of NMDA-receptor antagonist (Fowler et al., 2011). More recently, targeted knock-down of α3 nAChR subunit in either MHb or IPN was shown to produce similar increases in nicotine intake (Elayouby et al., 2021). Global overexpression of β4 nAChR subunit led to increased nicotine aversion, an effect reversed by selective expression of α5 nAChR subunit in MHb (Frahm et al., 2011). Together, these data indicate that nicotine acting on α5-containing, α3-containing, and β4-containing nAChRs facilitates nicotine-mediated excitatory transmission at MHb synapses in the IPN, which reduces nicotine-self administration. Importantly, our data demonstrate that cKO of VGLUT1 in the MHb led to increased nicotine self-administration, which is consistent with this framework and provides the first direct evidence that release of glutamate from cholinergic MHb projections to IPN inhibits nicotine self-administration.

### MHb heterogeneity contributes to diverse effects

The MHb is a heterogenous structure composed of several distinct cell types, each capable of releasing or coreleasing a variety of neurotransmitters or neuropeptides (Hashikawa et al., 2020; Wallace et al., 2020). For example, Fos data indicate that most MHb cell types are activated by foot-shock stress (Hashikawa et al., 2020). On the other hand, activity in dorsal MHb neurons, which are largely noncholinergic, may play a role in positive reinforcement and reward consumption (Hsu et al., 2014, 2016). Further, stimulation of glutamatergic septal inputs to MHb was anxiolytic, although different populations of MHb neurons were either inhibited or excited by this manipulation (Otsu et al., 2019). Thus, different MHb cell types appear to play opposing roles in mediating behaviors and affective states relevant to nicotine consumption.

Disruption of glutamate transmission from MHb to IPN could increase nicotine self-administration if this glutamate signal opposes nicotine reward or mediates aspects of nicotine aversion, although several lines of evidence favor the latter. For example, MHb projections to the IPN mediate negative affective behaviors such as anxiety, aversion, and the expression and extinction of fear memories (Fowler et al., 2011; Yamaguchi et al., 2014; Soria-Gómez et al., 2015; Pang et al., 2016; Zhang et al., 2016; Wolfman et al., 2018; Otsu et al., 2019). In mice undergoing nicotine withdrawal, optogenetic silencing of MHb inputs to IPN reduced marble-burying and increased time spent in the open arms of an elevated plus maze; microinjection of NMDA antagonist in IPN recapitulated this effect and was also shown to reduce somatic signs of withdrawal (Zhao-Shea et al., 2013, 2015). Therefore, the loss of glutamate release from cholinergic MHb projections in our study most likely led to increased nicotine consumption by reducing its aversive properties, but future studies are necessary to lend support to this conclusion. For example, while our study used a targeted genetic KO to demonstrate a novel role for MHb→IPN glutamate release in decreasing nicotine self-administration, directly measuring or manipulating glutamate release at this synapse during nicotine self-administration or conditioning assays would shed additional light and may help distinguish whether the glutamate signal is facilitating nicotine aversion or opposing nicotine reward.

### Cholinergic/glutamatergic cotransmission from MHb to IPN

Previous reports have detailed activation of the central IPN by MHb projections via fast glutamate-mediated currents, as well as by slower ACh-mediated currents (McGehee et al., 1995; Ren et al., 2011; Frahm et al., 2015). Histologic assessments of VGLUT1 and the vesicular ACh transporter (VAChT) have shown MHb axon terminals copositive for these transporters, and electron microscopy has identified vesicles at this synapse containing both vesicular transporters (Ren et al., 2011; Aizawa et al., 2012; Frahm et al., 2015). Our results are consistent with prior works showing that dorsal MHb, which is not cholinergic, projects to lateral IPN, while the cholinergic ventral MHb projects to central IPN (Herkenham and Nauta, 1979; Contestabile et al., 1987; Quina et al., 2017). Further, our experiments show presence of both *Slc17a7*/VGLUT1 and *Slc17a6*/VGLUT2 RNA transcripts in MHb, and that VGLUT2-expressing MHb neurons can also project to both lateral and central IPN, consistent with prior findings (Qin and Luo, 2009; Hashikawa et al., 2020; Wallace et al., 2020). In our VGLUT1 cKO animals, the residual glutamate-mediated oEPSCs in IPN are most likely facilitated by expression of VGLUT2. Nevertheless, cKO of VGLUT1 led to a large reduction in evoked glutamate currents and to decreased nicotine intake, although disrupting both vesicular glutamate transporters might produce an even larger effect which may be addressed in future studies.

Work by Frahm and colleagues used a similar cKO approach to disrupt ChAT expression in MHb and showed that this led to loss of nicotine withdrawal behaviors and loss of nicotine conditioned place preference (Frahm et al., 2015). Thus, despite both transmitters exerting postsynaptic actions that are primarily excitatory, glutamate and ACh release from MHb neurons appear to mediate different affective responses to nicotine, with acetylcholine release necessary for nicotine-associated reward, and glutamate release signaling nicotine aversion. This is perhaps more surprising given that these transmitters localize to an overlapping pool of synaptic vesicles and synergistic effects on vesicle filling are supported by data demonstrating that loss of ChAT/ACh reduces glutamate filling (Frahm et al., 2015), presumably because ACh uptake through VAChT dissipates the vesicular pH gradient and increases the vesicular membrane potential that VGLUT relies on for packaging glutamate (Hnasko and Edwards, 2012). And while we did not observe a reciprocal reduction in cholinergic transmission in the VGLUT1 cKO, this may be because of high variability in detection of cholinergic currents, or because of the presence of VGLUT2.

In conclusion, nicotine consumption is shaped by a balance of its rewarding and aversive actions, thus our understanding of the circuit mechanisms by which nicotine aversion is encoded is crucial for developing effective therapeutics for nicotine addiction. Our findings demonstrate a role for glutamate signals from MHb cholinergic projections to IPN in opposing nicotine self-administration and suggests that potentiating nicotine’s effect on this circuit could be a useful target for nicotine cessation therapies. Future work may also focus on dissecting the relative roles of glutamate, ACh or other cotransmitters in this circuit on other aspects of nicotine behavior or in mediating responses to other substances of abuse.

## Synthesis

Reviewing Editor: Alexxai Kravitz, Washington University in St. Louis Decisions are customarily a result of the Reviewing Editor and the peer reviewers coming together and discussing their recommendations until a consensus is reached. When revisions are invited, a fact-based synthesis statement explaining their decision and outlining what is needed to prepare a revision will be listed below. The following reviewer(s) agreed to reveal their identity: NONE.

Congrats on an excellent paper, thanks for submitting it to eNeuro!
